# An iron compass in the liver: superparamagnetic macrophages give homing pigeons a magnetic sense

**DOI:** 10.1038/s41392-026-03004-9

**Published:** 2026-10-21

**Authors:** Yun Liu, Jincheng Fang, Roman Sankowski

**Affiliations:** Institute of Neuropathology, Faculty of Medicine, University of Freiburg, Freiburg, Germany

**Keywords:** Innate immune cells, Cellular neuroscience

In a recent study published in Science, Lisowski et al. identified superparamagnetic, iron-loaded macrophages in the pigeon liver and showed that depletion of clodronate-sensitive phagocytic cells impaired homing under overcast skies, when solar cues were unavailable.^1^ Together, these observations point to a light-independent magnetoreceptive system outside the head and broaden current models of magnetoreception beyond receptors based on cryptochrome, magnetite, and ion channels in the eye and beak.^2^

**Fig. 1:**
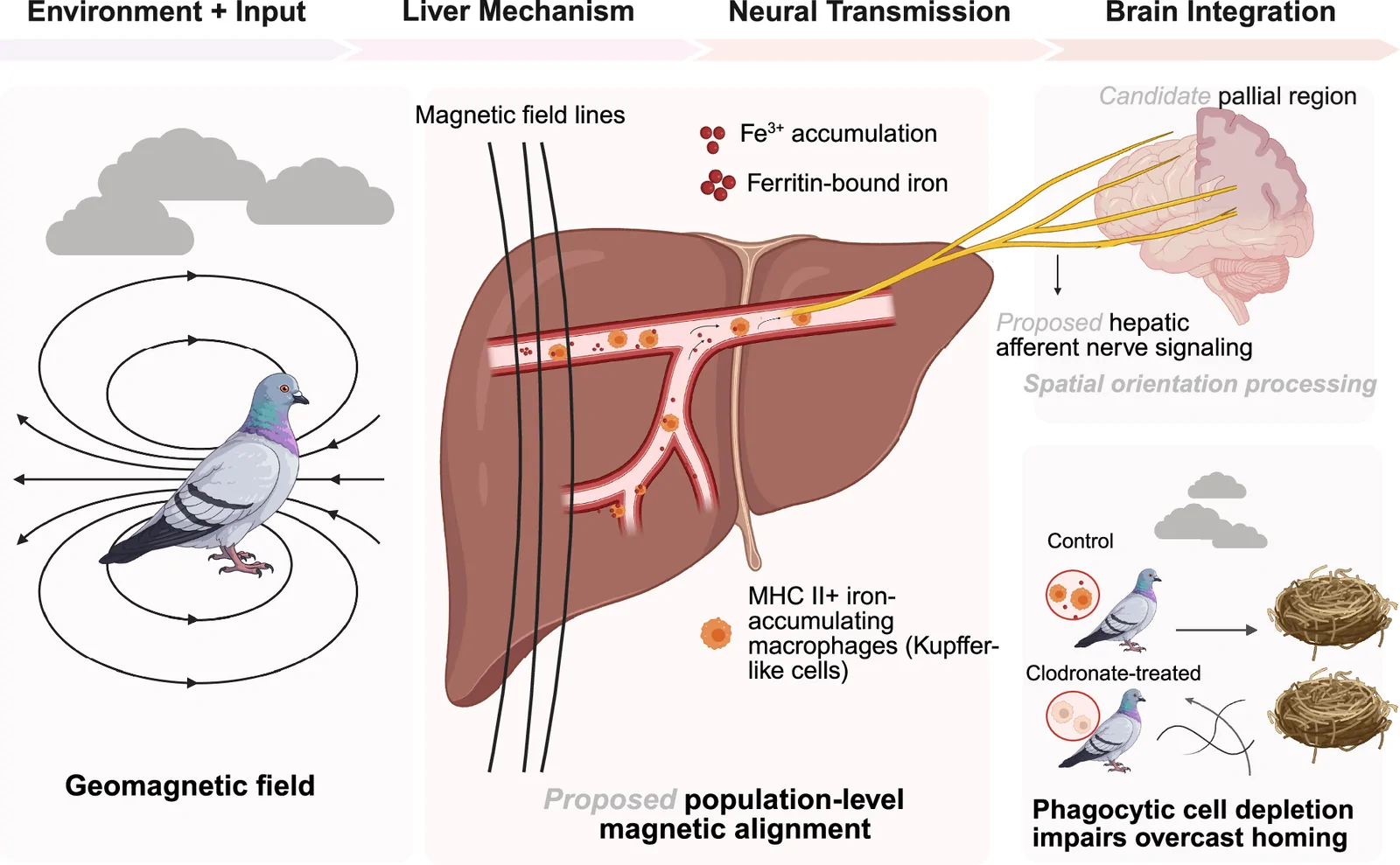
A liver-based route for magnetic orientation in homing pigeons. Hepatic MHC II+ macrophages store ferritin-bound iron (Fe^3+^) and are proposed to sense geomagnetic direction through proposed population-level magnetic alignment, potentially relaying this information to the brain via proposed hepatic afferent nerve signaling. Clodronate-sensitive phagocytic cell depletion impaired homing under overcast, but not sunny, conditions, consistent with impaired magnetic orientation under conditions lacking solar cues. Elements shown in gray italics denote proposed or candidate steps that were not directly demonstrated in the primary study, whereas experimentally supported elements are shown in black. The figure was created with BioRender.com

Magnetic measurements pointed the way (Fig. 1). Vibrating-sample magnetometry revealed a magnetic blocking transition near 12 K in liver and spleen, the signature of superparamagnetic nanoparticles, absent from muscle and beak. The measured signal could not be explained by isolated paramagnetic ferric iron, and low-temperature hysteresis indicated long-range magnetic order: not merely iron in the liver, but a biological nanomagnet. Prussian blue staining localized ferritin-bound iron to MHC II+ cells in hepatic sinusoids, abundant in liver, sparse in spleen, and absent from brain, eye, muscle, and beak.

Identifying the cells required a combination of histology and transcriptomics. Iron and MHC II colocalized, and sorted MHC II+ cells revealed a clear macrophage signature, together with enrichment of Spi-C, a transcription factor central to iron-handling red-pulp macrophages. Notably, no significant enrichment of dendritic-cell or B-cell signature was detected, an exclusion that matters because MHC II marks all three antigen-presenting lineages. Functionally, the cells phagocytosed dextran with high efficiency, consistent with an active macrophage identity. Together, these data indicate that the iron reservoir is not diffuse but cell-specific: the very macrophages that recycle iron from senescent erythrocytes accumulate ferritin in sufficient quantities to generate measurable magnetism. Because clodronate can also perturb neutrophils, the authors examined heterophils, the avian equivalent, and found them devoid of iron and of any magnetic blocking, supporting macrophages, rather than granulocytes, as the iron-containing candidate cells.

Behavioral experiments then provided the decisive test. Pigeons trained to home over a 19 km route were depleted of clodronate-sensitive phagocytic cells, under conditions favoring hepatic iron-loaded macrophages, and released under fully overcast skies, when solar and polarized-light cues are unavailable. Control birds returned within ~70 min, whereas treated birds dispersed in random directions and failed to home that day. Crucially, the same birds still homed under sunny conditions, arguing against impaired flight capacity, motivation, or health, and supporting a deficit in magnetic orientation under overcast conditions. The effect was robust across multiple overcast release days over two years. Anatomically, iron-loaded macrophages were found within ~2 μm of unmyelinated autonomic nerve fibers in the portal triads, a geometry compatible with proposed hepatic afferent nerve signaling. Clodronate treatment disrupted these macrophage–nerve associations while sparing the nerves themselves, supporting the possibility of a relationship between magnetic material and peripheral neural pathways, although direct signal transmission remains to be demonstrated.

These findings support macrophage-based magnetoreception as a potential fourth mechanism of magnetic sensing. Rather than specialized neurons or photochemical reactions, the authors propose that a population of ferritin-rich macrophages acts as a collective sensor. While the magnetic moment of a single cell is negligible, dipole–dipole coupling across many aligned cells could, in principle, amplify sensitivity to geomagnetic fields, echoing the ordered magnetosome chains of magnetotactic bacteria. The brief circling behavior observed after release may help align this distributed array before a stable heading is selected.

In 2012, iron-rich cells in the pigeon upper beak, long considered candidate magnetoreceptors, were shown to be macrophages rather than specialized sensory neurons, undermining the beak-based hypothesis.^3^ Lisowski et al. now reposition macrophages not as artifacts of that search, but as the sensory substrate itself, extending earlier observations that splenic red pulp macrophages are intrinsically superparamagnetic.^4^

Iron plays a central role in complex biological processes. It is essential for organismal survival by enabling oxygen transport through hemoglobin, and dysregulated iron turnover underlies hemochromatosis, the most common hereditary disease among people of European ancestry. Recent work from other research fields has identified altered iron turnover across various conditions. In prenatal development, altered iron turnover is required for physiological hypoxia adaptation. In cancer, iron homeostasis is central for cancer-associated hypoxia responses. This evidence highlights macrophage-mediated iron homeostasis as a key adaptation to physiological and pathological hypoxia.

Mechanistically, the case is not quite closed yet. Because clodronate depletes macrophages systemically, liver-specific causality remains to be demonstrated. How a slow ferritin-based magnetic alignment is converted into rapid neural signaling also remains unclear and will require quantitative biophysical modeling and independent replication. It is further unknown whether and how this system interacts with cryptochrome-based magnetoreception or other sensory modalities, including candidate vestibular–pallial circuits proposed to participate in magnetically induced neuronal activity in the pigeon brain.^5^

Even so, the study expands the landscape of biological magnetism. It suggests a light-independent receptor that could operate in animals lacking functional cryptochromes or living in darkness, and it raises a broader possibility: that tissue-resident macrophages are not only immune sentinels but peripheral sensory cells that report physical properties of the environment to the nervous system. If so, the homing pigeon’s compass may be the earliest example of an immune-cell-based sensory system, not the last.
